# Ependymoma and Carcinoid Tumor Associated with Ovarian Mature Cystic Teratoma in a Patient with Multiple Endocrine Neoplasia I

**DOI:** 10.1155/2014/712657

**Published:** 2014-04-10

**Authors:** Reed Spaulding, Houda Alatassi, Daniel Stewart Metzinger, Mana Moghadamfalahi

**Affiliations:** ^1^Department of Pathology, University of Louisville Hospital, 530 South Jackson Street, Louisville, KY 40202, USA; ^2^Department of Obstetrics and Gynecology, Division of Gynecologic Oncology, James Graham Brown Cancer Center, 529 South Jackson Street, Louisville, KY 40202, USA

## Abstract

Ovarian teratomas rarely undergo new neoplastic transformation and account for a small percentage of malignant ovarian germ cell neoplasms. Here we report a case of a 51-year-old woman with multiple endocrine neoplasia type I (MEN I) who was found to have an ependymoma and neuroendocrine tumor (trabecular carcinoid) associated with mature cystic teratoma of her left ovary. The ependymoma component displayed cells with round nuclei and occasional small nucleoli which were focally arranged in perivascular pseudorosettes and true rosettes. Rare mitoses were identified. No necrosis was present. Immunohistochemical staining was positive for S-100 and GFAP. The Ki67 proliferation index was very low (2-3%). In contrast, the endocrine tumor component was composed of small uniform cells with eosinophilic cytoplasm, round nuclei, and speckled chromatin. Immunohistochemical staining was positive for synaptophysin and focally positive for chromogranin. This rare case illustrates that MEN I may have an influence on the pathogenesis of ovarian teratomas as they undergo malignant transformation.

## 1. Introduction

Mature cystic teratomas (MCTs) are the most common germ cell tumors of the ovary [1]. Teratomas of the ovary originate from pathogenetically activated oocytes that can give rise to early embryonic structures arising from three germ layers: ectoderm, mesoderm, and endoderm [2].

Carcinoid tumors arising from the ovary are exceedingly rare and they are usually associated with MCT [1]. Primary neuroectodermal tumors are also known to rarely arise within the ovary, including ependymoma, astrocytoma, glioblastoma multiforme, ependymoblastoma, medulloblastoma, medulloepithelioma, and neuroblastoma [2]. These tumors are often classified as monodermal teratomas [3]. A PubMed search showed that only 19 cases of ovarian ependymoma have been reported in the literature and only one has been associated with a MCT. The latest case has been reported by Stolnicu et al. in 2011 [4]. In exceedingly rare cases, new neoplastic transformation of MCT can give rise to multiple separate tumor foci, each arising from its respective germinal epithelial layer.

Multiple endocrine neoplasia (MEN I) is a disease entity that is classically understood to cause parathyroid adenomas, enteropancreatic endocrine tumors, and pituitary tumors; however, it actually causes combinations of over 20 different endocrine and nonendocrine tumors [5]. Among some of the more uncommon neoplasms are thymic carcinoid tumors (2% of cases), bronchial carcinoid tumors (2%), and, very rarely, ependymomas (1%) [5]. Here we present a case of a patient with a long-standing history of MEN I, who developed a mature cystic ovarian teratoma with a mature epidermal and neural component. Interestingly, her tumor underwent new neoplastic transformation to include both ependymoma and neuroendocrine tumor components. This rare association has not been reported before and it may raise the possibility that MEN I might have influenced the pathogenesis of neoplastic transformation of the ovarian teratoma in this novel case.

## 2. Case Presentation

A 51-year-old woman with a history of MEN I was found to have a left ovarian mass on an outpatient computed tomography (CT) scan during a work-up for dysfunctional uterine bleeding. The patient had a long-standing history of recurrent primary hyperparathyroidism associated with her endocrine disease. She previously required multiple surgical resections of parathyroid adenomas, the first being in 1988, with additional resections in 2008 and 2012.

Following a diagnostic laparoscopy and hysteroscopy with dilation and curettage to address dysfunctional uterine bleeding, the patient was referred to the University of Louisville Department of Gynecologic Oncology by her primary care OBGYN. She presented with no clinical symptoms related to her incidental ovarian mass. She underwent a total robotic hysterectomy with bilateral salpingooophorectomy and cystoscopy. An intraoperative frozen section was sent for pathologic evaluation, which revealed a cystic teratoma. An immature component could not be completely ruled out, thus prompting the patient to be surgically staged.

**Figure 1:**
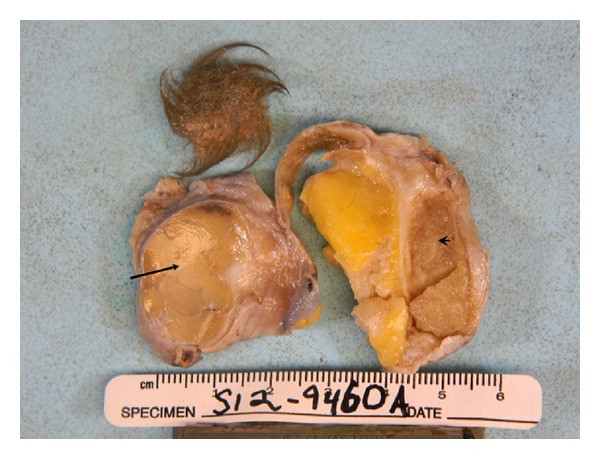
Gross photograph of the ovarian tumor, the arrow showing the neuroendocrine (carcinoid) component, and the arrowhead showing the ependymoma component.

Intraoperatively, sectioning through the left ovary revealed fatty to tan-pink and focally calcified cut surfaces, which were surrounded by a 5.5 × 3.0 × 1.0 cm uniloculated cyst containing coarse black hair. There were two discrete solid nodules arising from the cyst wall (Figure 1). One nodule had a soft gray-tan cut surface and it measured 3.0 × 2.5 cm. The other nodule was solid, firm, and tan-yellow on cut surface and it measured 2.5 × 2.0 cm. No evidence of necrosis was identified. The uterine body, right ovary, and bilateral fallopian tubes were unremarkable upon gross examination. Tissues were fixed in 10% neutral buffered formalin overnight and subsequently embedded into paraffin blocks. Sections were cut at 5-micron intervals and stained with hematoxylin and eosin. Additionally, a battery of immunohistochemical stains was ordered to better characterize the lesion.

**Figure 2:**
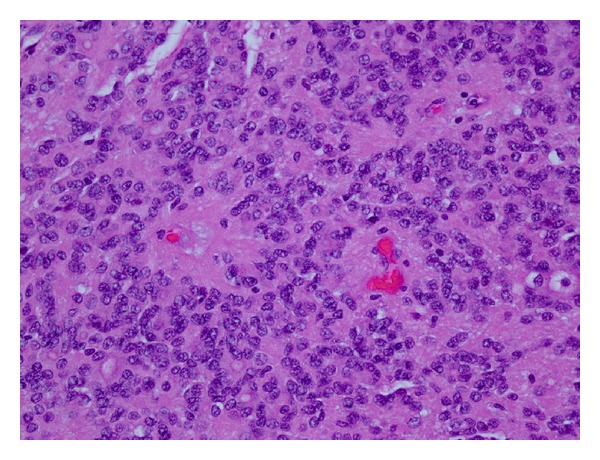
Histologic features of the ependymoma component with rosette formation.

Microscopic examination of the larger solid nodule revealed a heterogeneously cellular tumor with fibrillary background. The cells were uniform and round with occasional nucleoli. Frequent perivascular pseudorosettes as well as true rosettes with small to large tubules were noted (Figure 2). No necrosis, nuclear atypia, or evidence of vascular proliferation was identified. Mitotic figures were rare. The case was reviewed by an expert neuropathologist and special immunohistochemical stains were performed. A diagnosis of well-differentiated ependymoma was confirmed.

**Figure 3:**
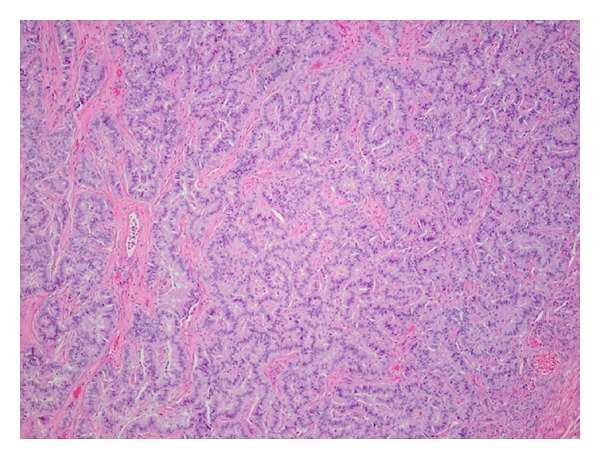
Neuroendocrine component (carcinoid) with trabecular tumor cell arrangement.

Microscopic examination of the second nodule showed a trabecular and cord-like arrangement of the tumor cells surrounded by dense fibromatous stroma. The tumor cells were uniform oval with finely dispersed chromatin (Figure 3). A diagnosis of neuroendocrine tumor (trabecular carcinoid) was confirmed by immunohistochemical stains. The final pathologic diagnosis was rendered as “ependymoma and trabecular carcinoid tumor arising from a mature cystic teratoma.” The tumor histology was well differentiated.

Apart from these two components, the remaining teratoma was comprised of skin and mature glial tissue. The tumor was completely confined to the left ovary. Nine lymph nodes as well as all biopsies from the surgical staging were negative for metastatic neoplasm.

**Figure 4:**
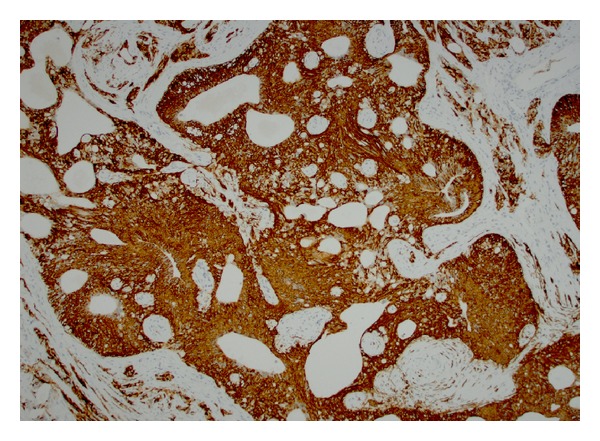
The ependymoma component stained positive for GFAP.

**Figure 5:**
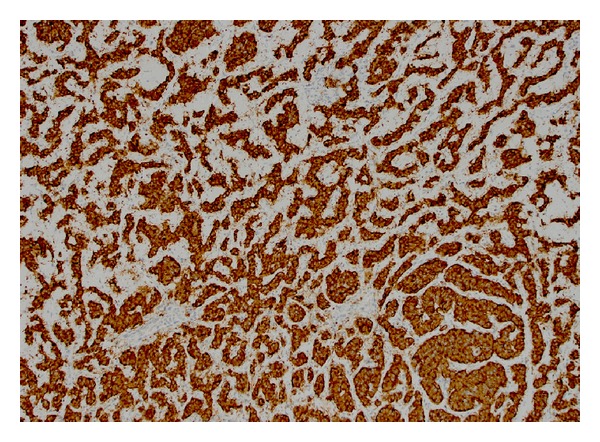
Neuroendocrine component (carcinoid) stained positive for synaptophysin.

**Figure 6:**
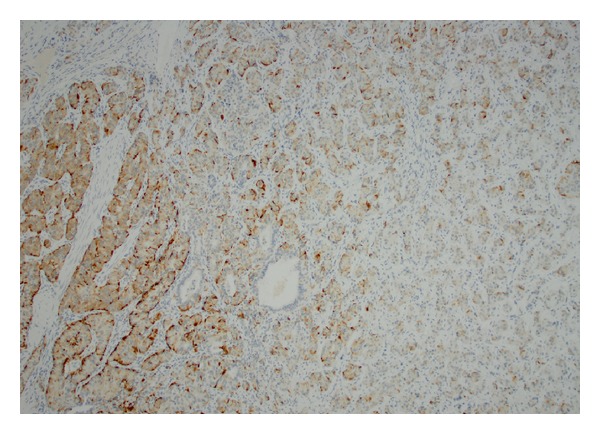
Neuroendocrine component (carcinoid) stained positive for chromogranin.

Immunohistochemical staining was performed on the tissue samples for pathologic confirmation of the diagnosis. The ependymoma component stained positive for S-100 and GFAP (Figure 4). Ependymoma cells were negative for synaptophysin, p53, and NeuN. Ki67 proliferation index was very low (2-3%). Tumor cells showed weak positive staining for estrogen receptor (ER) and weak to moderate staining for progesterone receptor (PR). In contrast, the neuroendocrine component stained positive for synaptophysin and focally positive for chromogranin (Figures 5 and 6). Carcinoid cells were negative for TTF-1 and thyroglobulin. Overall these immunohistochemical stains supported the diagnosis rendered.

Final pathologic staging was International Federation of Gynecology and Obstetrics (FIGO) stage IA. The patient was scheduled for a 6-month follow-up appointment. It was made clear to her that while her prognosis was good based on staging, close clinical follow-up would be needed. She has remained symptom-free since her surgery 12 months ago.

## 3. Discussion

Both MEN I and new neoplastic transformation of MCT are rare clinical entities. Our patient is exceptionally rare in that she was affected by both diseases. Based on the type of tumors that developed from the MCT seen in our patient, it raises the possibility that MEN I may alter the pathogenesis of MCTs as they undergo new neoplastic transformation. To our knowledge, this is the first reported case in the literature that would show synchronous association of ependymoma and neuroendocrine tumor with a MCT in a patient with MEN I.

In addition to the classic presentation of parathyroid adenomas, enteropancreatic endocrine tumors, and pituitary tumors that are most often associated with MEN I, carcinoid tumors are rarely encountered. They can present in the bronchi, gastrointestinal tract, pancreas, and thymus [6]. The prognosis of these tumors differs based upon their anatomic location. Thymic carcinoid tumors developing in MEN I patients are particularly aggressive and cause an increased risk of death, whereas bronchial carcinoid tumors have not been shown to increase mortality [6]. While there is no universal agreement on the time interval to screen for these neoplasms, some authors suggest radiologic imaging every 1-2 years [6, 7].

The development of ependymomas in patients with MEN I is an extremely rare entity. As of 2010, there were only four reported cases in the literature [8]. Three of those cases were spinal ependymomas. The case reported in 2010 was an intracranial grade II ependymoma developing in a 44-year-old female patient with MEN I [8]. Her tumor was resected in 2003 but recurred the following year with higher grade histology. She expired that same year.

The most recent case of spinal ependymoma was recently reported in a 53-year-old man with MEN I [9].

The MEN I gene is a putative tumor suppressor gene located on chromosome 11q13 [8]. Tumor development in MEN I patients follows the “two hit” hypothesis, where patients affected by familial MEN I inherit one wild-type and one mutant allele [8]. Later in life, tumors can then develop in various tissues after mutation of the remaining wild-type allele occurs. In 1997, Giraud et al. documented the first MEN I-related ependymoma with loss of heterozygosity in the MEN I gene, implicating that this gene may be involved in the tumorigenesis of ependymomas [10].

MCTs, by definition, can develop into tissues derived from any of the three embryonic germ cell lines. The secondary malignancy that develops in these fairly common neoplasms will be influenced both by the patient's underlying genetic mutations and environmental factors. Our patient developed two separate tumors in her MCT that have both been documented to occur in association with MEN I. To our knowledge, this is the first case that would illustrate a potential relationship between MEN I and the subsequent components of a MCT following malignant transformation. While the case is exceedingly rare, it raises the following question: “do the genetic alterations associated with MEN I predispose women with MCTs to undergo malignant transformation at higher rates?” Such knowledge could have a significant impact on the screening algorithms of MEN I patients for MCTs. The data is currently limited and further study is required.
